# Help-Seeking Situations Related to Visual Interactions on Mobile Platforms and Recommended Designs for Blind and Visually Impaired Users

**DOI:** 10.3390/jimaging10080205

**Published:** 2024-08-22

**Authors:** Iris Xie, Wonchan Choi, Shengang Wang, Hyun Seung Lee, Bo Hyun Hong, Ning-Chiao Wang, Emmanuel Kwame Cudjoe

**Affiliations:** School of Information Studies, University of Wisconsin-Milwaukee, Milwaukee, WI 53211, USA; wchoi@uwm.edu (W.C.); shengang@uwm.edu (S.W.); lee649@uwm.edu (H.S.L.); hongb@uwm.edu (B.H.H.); wang424@uwm.edu (N.-C.W.); ekcudjoe@uwm.edu (E.K.C.)

**Keywords:** blind and visually impaired users, visual interactions, help-seeking situations, mobile platforms, digital libraries, design recommendations

## Abstract

While it is common for blind and visually impaired (BVI) users to use mobile devices to search for information, little research has explored the accessibility issues they encounter in their interactions with information retrieval systems, in particular digital libraries (DLs). This study represents one of the most comprehensive research projects, investigating accessibility issues, especially help-seeking situations BVI users face in their DL search processes. One hundred and twenty BVI users were recruited to search for information in six DLs on four types of mobile devices (iPhone, iPad, Android phone, and Android tablet), and multiple data collection methods were employed: questionnaires, think-aloud protocols, transaction logs, and interviews. This paper reports part of a large-scale study, including the categories of help-seeking situations BVI users face in their interactions with DLs, focusing on seven types of help-seeking situations related to visual interactions on mobile platforms: difficulty finding a toggle-based search feature, difficulty understanding a video feature, difficulty navigating items on paginated sections, difficulty distinguishing collection labels from thumbnails, difficulty recognizing the content of images, difficulty recognizing the content of graphs, and difficulty interacting with multilayered windows. Moreover, corresponding design recommendations are also proposed: placing meaningful labels for icon-based features in an easy-to-access location, offering intuitive and informative video descriptions for video players, providing structure information about a paginated section, separating collection/item titles from thumbnail descriptions, incorporating artificial intelligence image/graph recognition mechanisms, and limiting screen reader interactions to active windows. Additionally, the limitations of the study and future research are discussed.

## 1. Introduction

Digital libraries (DLs), a type of information retrieval (IR) system, offer structured digital collections designed to support information access, management, and dissemination [1]. However, the sight-centered and complex design of current DLs—such as layered navigation involving categories and collections and detailed metadata for individual items—often leads blind and visually impaired (BVI) users who rely on screen readers to encounter help-seeking situations. Help-seeking situations refer to situations where users need additional assistance from systems or other human agents to accomplish specific tasks or goals [2]. Help-seeking situations reflect not only accessibility and usability issues of IR systems but also user experience, highlighting the gap between system design and users’ diverse needs.

Visual interactions refer to the ways users engage with non-text interface design features/elements, non-text document content, and sight-centered page layout and site structure that require visual recognition. These interactions can be more challenging for BVI users because they may require more detailed text-based descriptions, the use of advanced screen reader features (e.g., image description and screen recognition), or other assistive technologies (e.g., braille displays). Furthermore, on a mobile platform (e.g., iOS or Android) that enables mobile devices to run applications [3], visual interactions are likely more challenging due to the often condensed or cramped interface designs of DLs resulting from the small screen sizes of mobile devices.

Given the widespread adoption of mobile devices among individuals with disabilities [4] and the prevalence of visual impairments [5], it is important to examine help-seeking situations related to visual interactions on mobile platforms and devices. Prior studies have examined some help-seeking situations associated with visual interactions encountered by BVI users in both desktop and mobile environments [2,6,7,8,9]. Although these studies provided useful insights about improving accessibility and usability of IR systems, including DLs, for BVI users, there is a need for further research to examine typical and unique help-seeking situations across diverse mobile platforms. As part of a larger project to develop comprehensive design guidelines for DLs to support BVI users using screen readers on two mobile platforms and four popular types of mobile devices—Android phone, Android tablet, iPhone, and iPad—this paper presents selected findings aimed at addressing the two research questions (RQs):

RQ1: What are the overall categories of help-seeking situations encountered by BVI users in the mobile environment and their distributions?

RQ2: What are the typical types of help-seeking situations related to visual interactions that BVI users encounter in the mobile environment?

While RQ1 focuses on the overall categories of all the help-seeking situations encountered by BVI users in the mobile environment, RQ2 emphasizes the specific types of help-seeking situations associated with visual interactions. Moreover, RQ1 also investigates the distribution of categories of all help-seeking situations. The two RQs focus on different aspects of these situations.

## 2. Literature Review

### 2.1. BVI Users’ Help-Seeking Situations Related to Visual Interactions

BVI users’ help-seeking situations, especially those related to visual interactions, have been examined in both desktop and mobile environments. Much research has been focusing on BVI users’ help-seeking situations related to visual interactions in the desktop environment [8,9,10,11,12,13,14,15]. Compared with the desktop environment, less research has been conducted in the mobile environment.

In the mobile environment, BVI users often encounter help-seeking situations associated with features/elements that constitute visual components of user interfaces. Specifically, BVI users often encounter challenges related to locating and accessing features/elements due to labeling issues (e.g., missing, improper, or inadequate labels) in mobile websites and applications [7,16,17,18,19,20,21,22]. For example, Wentz and co-authors [21] discovered that BVI users had difficulty accessing features/elements of banking and finance systems on mobile platforms. Similarly, Nair et al. [22] noted that BVI users had difficulty locating checkout or shopping buttons while using mobile food delivery applications. In mobile DLs, research shows that difficulty locating a feature was the second most frequent help-seeking situation for BVI users on the mobile website of a DL, including difficulty locating a toggle-based feature; in addition, understanding or using a specific feature was also identified as a help-seeking situation in mobile DLs [2]. Some studies examined relevant situations in a more general context. For example, difficulty identifying a button was among the most frequent problems for blind users in the mobile environment [7]. Moreover, BVI users were found to have difficulty fully using existing accessibility features (e.g., magnification, color inversion, and color contrast) on their smartphones or supported gestures in mobile applications due to their unawareness of the existence of such features or gestures [6,19].

Moreover, BVI users are likely to encounter help-seeking situations related to accessing the content of visual documents (e.g., images and graphs). These situations occur mainly due to a lack of alternative (ALT) text or descriptions or inconsistent ALT text for visual documents [7,16,22,23,24,25]. For example, Wang et al. [25] found that blind and low-vision users had difficulty understanding images of food products. Notably, difficulty accessing the content of visual documents was found to be the most frequent situation in the mobile website and the mobile app of a DL [2]. Accessing images is particularly challenging for BVI users when visual-related features (e.g., zooming features and image description features) are missing [6,26]. BVI users often have difficulty accessing the content of graphs, as discussed by Alajarmeh [16], who mentioned VI users’ difficulties with graphic content, images, and other visualizations. According to Radcliffe et al. [27], participants with visual impairments had trouble accessing graphic representations of data when using mobile health applications due to inconsistent ALT text. In addition to images and graphs, VI users also reported difficulty accessing or reading PDF files on their phones [26,28] and difficulty understanding the content of visual documents when too much information was presented on a mobile interface [7].

Some help-seeking situations are related to system structures, particularly layers and layouts, which are associated with visual interactions. For example, Milne et al. [18] found that BVI users had difficulty comprehending the overall structure and layout of mobile health applications due to unclear element labels. Similarly, Kim et al. [29] found that VI users had difficulty memorizing the interface layout of a mobile application. In mobile DLs, according to Xie et al. [2,30], BVI users may have difficulty orienting DLs’ overall structures and layouts due to their layered and complex design (e.g., complex information presentation). Moreover, Radcliffe et al. [27] pointed out that people with disabilities, including BVI users, had difficulty using inconsistent mobile interface displays. Difficulty with navigation is another common type of help-seeking situation related to structures [7,16,19]. For example, BVI users have difficulty navigating on mobile devices due to missing navigation elements (e.g., a “home” or a back button) [16]. BVI users also have difficulty interacting with inaccessible pop-up windows. Alamri et al. [31] found that students felt trapped in pop-up windows that screen readers could not read, could not understand the goal of pop-up windows, and could not understand how to interact with those windows.

### 2.2. Design Issues and Solutions

The likelihood of BVI users encountering features and elements that require visual interaction has increased due to sight-centered interface design. Researchers have discussed the importance of labeling essential page elements, such as links or buttons, particularly for image-based elements [2,22,32]. Fok et al. [32] pointed out that there are frequent instances of missing labels for non-text interface design elements and a lack of improvement over time in their longitudinal analysis of applications. To address this issue, they suggested that developers encourage the reuse of non-text elements and their labels when coding. This would help ensure a cohesive and consistent use of labels for the same functions, thereby preventing developers from omitting labels for visual elements and using inconsistent labels for the features with same functions. Nair et al. [22], in their study on food delivery applications, discovered missing and inappropriate ALT text for non-text interface design elements from their interviews and expressed the importance of providing adequate ALT text for visual design elements. Advanced technology, such as artificial intelligence (AI), has been studied to deal with the challenges of visual interactions [33,34]. For example, Jain et al. [33] suggested combining image processing AI and crowdsourcing to enhance accuracy in suggesting or labeling unlabeled buttons within mobile applications. Studies on improving text-to-speech systems have been conducted to increase comprehension of online content with emojis for BVI users [35,36].

For visual content, previous studies have emphasized the importance of providing adequate ALT text for BVI users interacting with images. BVI users who rely on screen readers can access images when a sufficient description of an image is provided with ALT text to convey the visual elements or information included in an image. Providing adequate ALT text, therefore, determines the ease of access to the content of images. Concerning document accessibility, Hovious and Wang [37] investigated the accessibility of library of information science journal papers within aggregated databases. They emphasized the need for all stakeholders to ensure document accessibility. This includes providing HyperText Markup Language (HTML) text and accessible PDF documents, ensuring proper tagging to signify document structure, and providing ALT text for images and figures.

Furthermore, one of the visual interactions encountered by BVI users is data visualization, which is designed to help people understand complex data by making it into an understandable format. However, without accessibility considerations, data visualization can pose difficulties for BVI users. Researchers have investigated methods that allow visualized data to be usable by BVI users [38,39,40]. Kim et al. [40] introduced a preliminary model for accessible visualization design. For visualization to be accessible, a notification of its existence is needed, followed by a summary of the data. Moreover, upon request, detailed information regarding the presented data should be accessible to users, and contextual cues about the data should be provided for exploring the data visualization. In a different approach, Singh and Joshi [41] proposed a graph visualization tool to help BVI users understand the concept of line charts. The tool allows users to adjust x and y values with sliders to identify chart trends. It provides a tactile learning tool for BVI users to grasp a fundamental understanding of charts by modifying them directly.

Navigating and understanding a page is vital when users interact with IR systems and applications. Therefore, researchers have explored various methods to facilitate the understandability of systems and page structures. For example, Duarte et al. [42] drew attention to the vision-based page segmentation of a webpage. They aimed to create an environment where BVI users could understand this segmentation by providing an automated role identification algorithm to identify menus on a page. In their definition, a menu is a set of links with roles designed to navigate users to assigned sections or pages; breadcrumbs, slideshows, and carousels are examples of menus. They experimented with developing an algorithm to detect and analyze various element roles to filter elements with navigation roles and present them as a menu that assists navigation. Therefore, BVI users would be able to detect grouped navigation-related links quickly.

Furthermore, studies have also emphasized the need to design clear and simple structures and navigation mechanisms for BVI users as they engage with interfaces sequentially using assistive technologies [2,7]. For example, Xie et al. [2] recommended a shallower and broader structure for simplifying complex structures. Nair et al. [22] proposed adhering to a similar interface structure across different applications to minimize users’ need to familiarize themselves with other applications. Zaina et al. [43] suggested incorporating an option labeled “more” or implementing a scrollable navigation tab bar for extensive navigation options, allowing users to access additional options with a digestible number of options at a time.

Even though previous research has identified BVI users’ help-seeking situations in the desktop DL environment, sparse research has examined BVI users’ help-seeking situations in the mobile DL environment. Only our prior pilot study [2] reported help-seeking situations faced by BVI users in their interactions with DLs, but that study only involved one DL and used one device type. The current study expands on the pilot study and concentrates on the help-seeking situations that BVI users faced in their visual interactions with six DLs across four types of devices on both Android and iOS platforms. Moreover, while previous research has investigated diverse aspects of design recommendations to provide a more accessible environment, there is a gap in research on design recommendations for IR systems, such as DLs, in the mobile environment. Therefore, there is a need to investigate help-seeking situations to develop corresponding recommendations.

## 3. Materials and Methods

### 3.1. Sampling

Since the study involved BVI users as a group of vulnerable participants, approval was sought and granted by the Institutional Review Board. The study recruited 120 BVI participants across the United States, with 30 in each of 4 groups representing different types of mobile devices (iPhone, iPad, Android phone, and Android tablet). All BVI participants relied on screen readers to interact with mobile devices. Each participant used the default screen reader of their devices: VoiceOver for the iPhone/iPad groups and TalkBack for the Android phone/Android tablet groups. A variety of methods were used to recruit BVI users. Most importantly, the recruitment flyer was distributed through the National Federation of the Blind. In addition, the research team attempted to find online communities of BVI users using IR systems and posted recruitment messages in such communities. Moreover, snowball sampling was used to find more BVI users, especially for the Android groups. Participants who had finished the study were encouraged to share the research project information with their BVI peers who they thought might be interested in the study.

BVI people needed to fulfill certain inclusion criteria for the study. Eligible participants were required to (1) be 18 years old or older, (2) rely on a screen reader to access the Internet, (3) have at least three years of experience in using mobile devices to search for information, (4) feel comfortable verbalizing in English, and (5) be willing to install Microsoft Teams on their mobile devices.

Table 1 provides the demographic information of the 120 BVI participants. Most participants (81%) were under 50, and only 19% were 50 or above. The numbers of male and female participants were almost equal, including 60 female participants, 59 male participants, and 1 non-binary participant. Regarding race, 45% of participants were White, 20% were Black, 16% were Asian or Pacific Islander, and 10% were Hispanic or Latino. Regarding education, 66% of the participants had a bachelor’s or master’s degree, and 34% had a high school or associate degree. The vision condition of participants indicates that 67% of the participants were blind. The rest were visually impaired users who relied on screen readers to interact with DLs on mobile devices.

### 3.2. Data Collection

Six DLs were selected for the study based on their diverse domains (e.g., history, art, science, and museum collections), diverse formats (e.g., text, image, video, and mathematical formulas), diverse types of hosting organizations, and different DL types (e.g., stand-alone, federated). These were the Library of Congress Digital Collections (LoC), Digital Public Library of America (DPLA), HathiTrust, ArtStor, OER Commons, and the National Museum of African American History and Culture. Each participant was assigned to use two DLs, including LoC and one of the other five DLs.

The larger research project employed multiple data collection methods, including pre-questionnaires, think-aloud protocols, transaction logs, post-system interviews, and post-search interviews. This study mainly used pre-questionnaires, think-aloud data (textual transcripts), and transaction log data captured by screenshots and video recordings.

**Figure 1:**
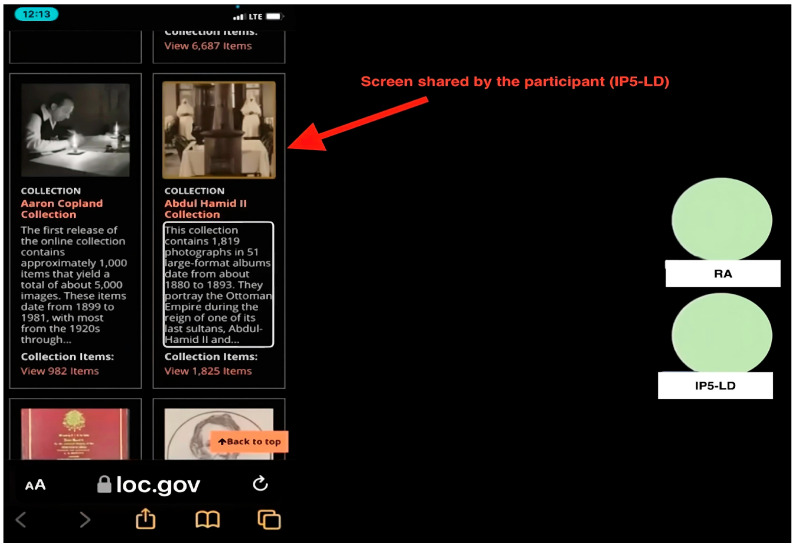
An example of Microsoft Teams meeting sessions.

The following is the data collection procedure. First, participants completed the pre-questionnaires to provide background information (e.g., demographic information, information search skills, and experience of using DLs). Second, each participant joined an online meeting via Microsoft Teams using their own devices in their natural settings to work on three tasks, including one orientation task and two search tasks (a specific search and an exploratory search) on each of the two DLs. Before the search tasks, they completed one 10-min orientation task to familiarize themselves with the assigned DLs. Table 2 presents three task types and associated examples. Third, think-aloud protocols were used when participants worked on the tasks. Specifically, they were instructed to talk continuously about their thoughts and actions in relation to their interactions with each DL, specifying their intentions for their actions, the problems they encountered, and their solutions. At the same time, transaction logs were recorded. We incorporated a screenshot of a participant’s shared screen to help readers understand a participant’s study setting. Figure 1 presents the Microsoft Teams meeting interface. Fourth, post-system interviews were conducted after each DL to solicit information about participants’ experiences and perceptions of the accessibility and usability of each DL. A post-search interview was conducted at the end of their participation to gather a final assessment of the two selected DLs. Participants’ interactions with DLs during tasks and interviews were recorded using Microsoft Teams, and video and transcripts were exported for analysis.

### 3.3. Data Analysis

Qualitative data from the transcripts were examined to address the proposed RQs. Moreover, videos were constantly being checked. Relevant screenshots were taken to help illustrate identified help-seeking situations. Open coding was used for data analysis, representing the process of breaking down, examining, comparing, conceptualizing, and categorizing textual transcripts [44]. The paper presents the seven types of help-seeking situations related to visual interactions on four types of mobile devices. Table 3 presents the coding scheme of these situations associated with features/elements, content, and structure. To avoid repetition, examples of these situations are presented in Section 4. Inter-coder reliability was 0.81 based on Krippendorff’s Alpha [45,46], indicating a good level of agreement among coders. Multiple sessions of group discussions were held to scrutinize the identified help-seeking situations and associated design recommendations. Descriptive data analysis was used to present the frequency distribution of different types of help-seeking situations (e.g., features/elements, content, and structure) based on the preliminary coding results.

## 4. Results

### 4.1. Categories of Help-Seeking Situations

Four categories of help-seeking situations were identified: features/elements, content, structure, and others. The features/elements category involves situations that arise from challenges associated with available features and interface elements on a page, such as buttons, banners, and carousels. The situations in this category include situations such as difficulty finding a toggle-based search feature, difficulty interpreting feature labels, and difficulty exiting out of a feature. The content category involves situations that arise from challenges associated with accessing or interacting with items and collections. The situations in this category include situations such as difficulty recognizing the content of graphs and difficulty assessing the format of an item. The structure category involves situations that arise from challenges associated with the layout or structure of a page or system. The situations in this category include difficulty understanding the page structure, difficulty identifying the current location or path, and difficulty understanding the digital library structure. The other category includes situations where users face difficulties unrelated to features/elements, content, or structure, such as compatibility issues.

**Figure 2:**
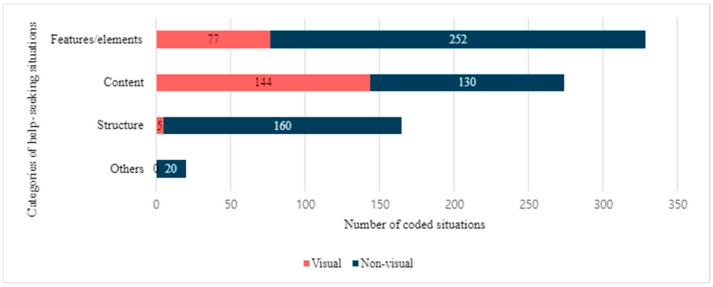
Distribution of different categories of help-seeking situations.

As a result of the open coding analysis, it was found that participants experienced a total of 788 instances of help-seeking situations. On average, each participant encountered approximately seven help-seeking situations while completing their tasks. The features/elements category (*n* = 329) was the most frequently coded, constituting approximately 41.8% of the total coded situations instances, with 77 instances (23.4%) related to difficulties in visual interaction (Figure 2). The content category (*n* = 274) accounted for about 34.8% of the coded help-seeking situations instances, with 144 (52.6%) associated with visual interactions. About 20.94% of the help situations are associated with the structure category (*n* = 165), with only five instances (3.03%) associated with visual interactions.

### 4.2. Typical Help-Seeking Situations Related to Visual Interactions in Mobile Environments

Seven typical types of help-seeking situations were addressed on four types of mobile devices using participant quotes. In the following examples, each participant ID begins with the specific device group (IP, ID, AP, AT), followed by the subject number within the group, and ends with two letters (AL, DL, HL, ML, OL) representing the abbreviations of two DLs assigned to the participant. For example, IP5-LD indicates that the participant is in the iPhone group (IP), has the subject number 5 within the group, and has been assigned to the Library of Congress and DPLA (LD).

#### 4.2.1. Features/Elements: Difficulty Finding a Toggle-Based Search Feature

**Figure 3:**
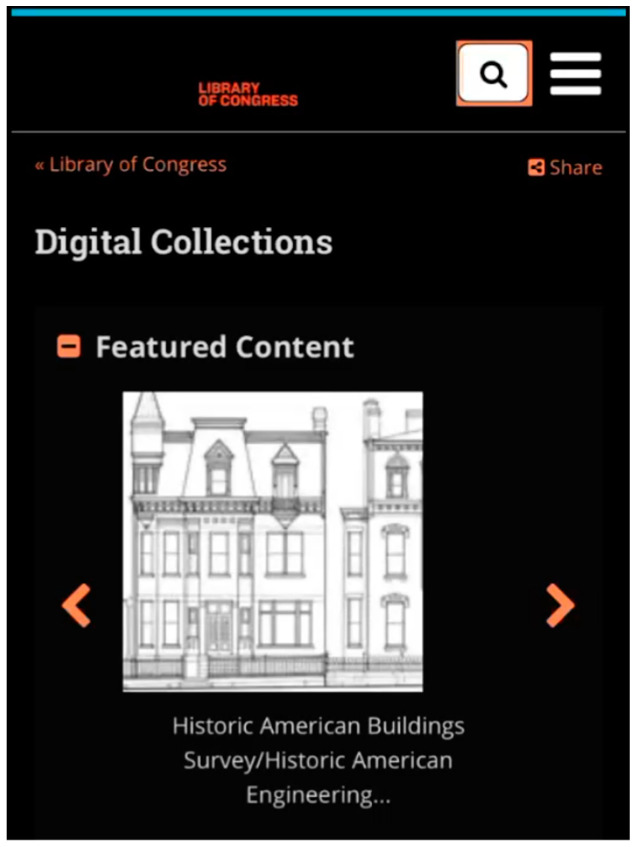
An example of difficulty finding a toggle-based search feature.

This situation occurs when BVI users have difficulty finding a search box due to the search toggle button and the multiple steps needed to find the search box. The non-text interface elements, such as buttons with visual cues but without understandable labels, pose a challenge for BVI users. IP5-LD expressed her confusion in understanding the label “search toggle” during her first encounter with one of the search features in the DL (Figure 3). During her orientation task, when she was scanning the page to explore the DL, she was initially uncertain whether she was interacting with a search feature:

“I don’t know if that’s like a search. Ohh, I guess it’s a search, but it’s not really a button. Let’s see a search toggle. I don’t really understand what that means. Ohh so I guess the search toggle brings up the search bar and then then you have a text field that wasn’t … really sure what search toggle meant. I’ve never heard that before, so now I’m in the search field. Umm, OK cool. So let me just try. Uh. I don’t know. I just wanna see how it works when you search for something.”

(IP5-LD)

**Figure 4:**
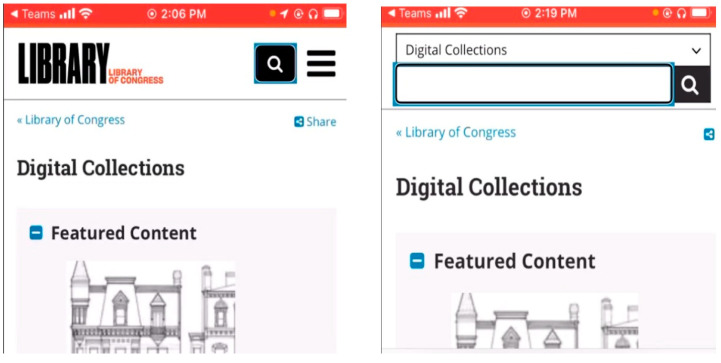
Another example of difficulty finding a toggle-based search feature.

Moreover, a hidden search field caused by responsive design creates additional steps needed to locate the search field, adding to complications that hamper easy access to the search field. For example, IP29-LH tried to find the search feature to conduct her search, but it was represented as a visual icon of a magnifying-glass labeled as search toggle. The two-step search toggle created an inconvenient environment for her to access the search field easily. She needed to interact with the magnifying glass icon first, which would then present her with the search field (Figure 4):

“I wish that the search field was not hidden behind the button because if I’m learning of this page… But if I’m loading up this page and I want to jump straight to the search field. … See, I can’t find the text field.”

(IP29-LH)

This situation arises from inappropriate labeling and a lack of intuitive design in the feature. In the previous examples, the search field was changed to a search toggle by the responsive design to fit the screen due to the space limitations of mobile devices. However, as indicated by IP5-LD, terms such as “search toggle” might not effectively convey the feature’s function to participants. Furthermore, IP29-LH emphasized that exposing the search field on a page helps BVI users locate and access the search feature to enter a query.

#### 4.2.2. Features/Elements: Difficulty Understanding a Video Feature

**Figure 5:**
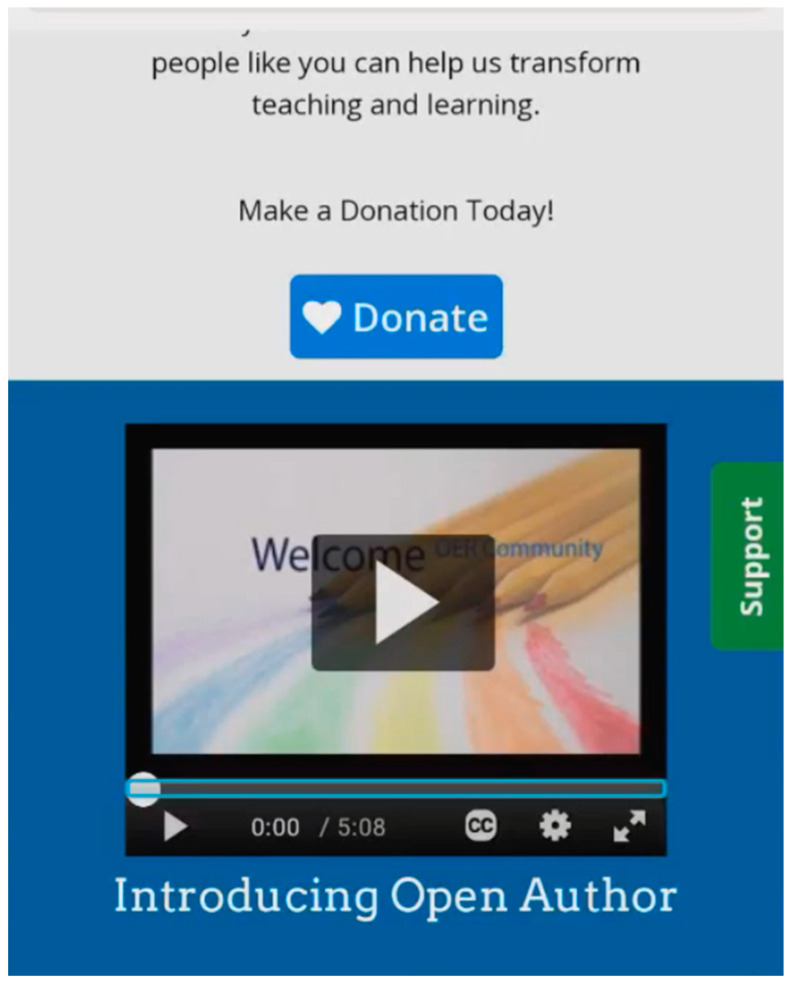
An example of difficulty understanding a video feature.

This situation occurs when BVI users face difficulty interpreting the functionality of or executing a video-related feature due to inappropriate labeling. Visual cues, such as the play button or video player progress bar, do not provide the necessary information for BVI users to understand their functionality. Therefore, without clear and comprehensive labels that assist BVI users in understanding the functionality of video-related features, a challenging environment is created, preventing users from fully utilizing the feature and accessing video content. For example, AP14-OL expressed his confusion upon encountering a video on a DL page. While swiping through the DL during his orientation task, he attempted to interact with the video. However, he indicated that the labels associated with the video-related features were inadequate in helping him understand how to utilize the feature (Figure 5).

“I’m noticing is it’s a little bit verbose, so it’s not properly tagged. So, it’s telling me and, you know, a bunch of numbers and what have you. It doesn’t really make sense from a screen reader point of view. So, OK, it’s telling me that there is a video thumbnail. OK, there is a button for play clip. Alright, so there is a slider. I’m not sure if that works or not, but I’ll just flick past that….”

(AP14-OL)

The absence of clear and comprehensive labeling results in confusion and presents a challenge for the participant to predict how to utilize the features presented to him. Sighted users can easily interact with the play button designed as a triangle and video progress bar, as these visual cues are intuitive. However, these cues are not accessible to users with visual impairments, making it difficult for them to understand how to use these features. As mentioned by AP14-OL, the available tags are meaningless and nonsensical without proper context, as they only consist of numbers and text.

#### 4.2.3. Features/Elements: Difficulty Navigating Items on Paginated Sections

**Figure 6:**
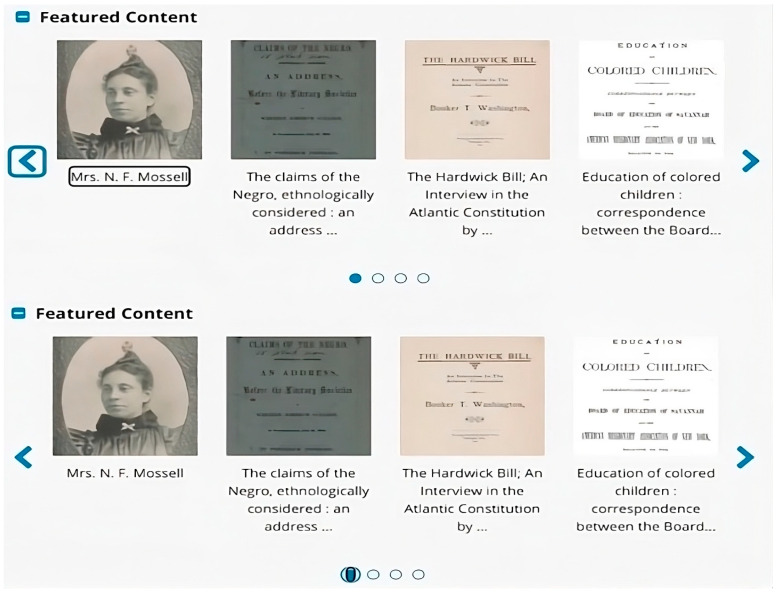
An example of difficulty navigating items on paginated sections.

This situation occurs when BVI users have difficulty selecting an item on paginated sections due to inappropriate labels of active elements. Paginated sections in DLs often consist of visual documents (e.g., images and scanned documents) across multiple pages instead of one single section. When interacting with visual items on a paginated section, BVI users need to use tabs or arrows to navigate items displayed on each page. As shown in Figure 6, the “Feature Content” section is paginated: in the first screenshot, the focus was on the label of the first item displayed on the first page; in the second screenshot, the focus was on the first of the four tabs at the bottom of the paginated section. According to ID17-LO, he could tell that there were tabs in the paginated section and that the first tab was selected. However, he was unable to determine what those tabs were in the “Feature Content” sections during the orientation task, which led to his confusion about the functions of the tabs.

“The tabs inside of the collection are also not accessible, so there are tabs over here. There’s one that’s selected, and it’s an unlabeled tab. The second one is not selected, third one, fourth one. At least I know that there are tabs here, but it doesn’t tell me what those tabs are.”

(ID17-LO)

As ID17-LO mentioned, this help-seeking situation occurred to him due to the missing labels of active elements (e.g., tabs) in the “Featured Content” section. The four tabs at the bottom of the “Featured Content” section are associated with the four separate pages that display different featured collection items. Without appropriate labels of these tabs to indicate their order (e.g., 1 out of 4 pages), ID17-LO was confused about the tabs and had no clear idea of the overall structure of the paginated section.

#### 4.2.4. Features/Elements: Difficulty Distinguishing Collection Labels from Thumbnail Descriptions

**Figure 7:**
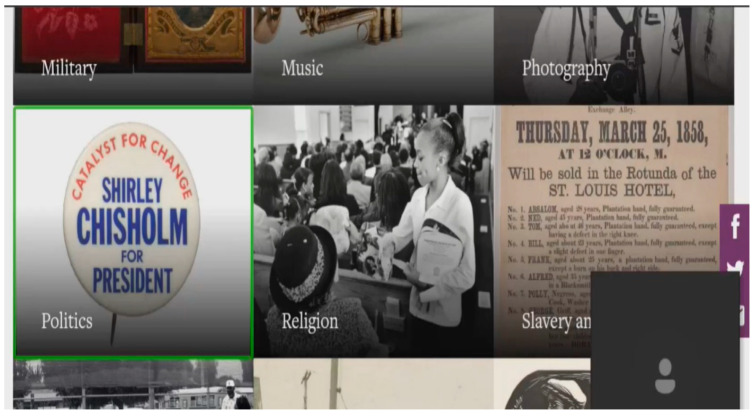
An example of difficulty distinguishing collection labels from thumbnail descriptions.

This situation occurs when a thumbnail and a label associated with a collection title are read together, posing a challenge for BVI users to comprehend and interact with the collection effectively. In an attempt to provide a description of the image associated with the collection, the ALT text of a collection thumbnail and the collection title are read out as one label to a screen reader. For example, AT24-ML expressed his confusion while browsing collections. In the orientation task, he was asked to explore the features and functions of the DL to familiarize himself with the environment. During this task, he encountered the list of collection topics through the “gallery view” option on the page. However, while going through the list of collections, he found it hard to recognize the collection titles as the descriptions were narrated alongside the title without clear differentiation, leaving him guessing the title (Figure 7).

“It doesn’t act like there’s a break between the description and what the category is…this one says white button with blue and red lettering politics explore so politics. But it’s not pausing to tell me it’s politics. It just runs it all through like it’s one word or like it’s one sentence. It’s so it’s some of these, it’s actually hard to tell what the category is because the category name fits into the sentence.”

(AT24-ML)

As illustrated by the participant, this situation arises because the design fails to separate the collection label from the thumbnail description. BVI users cannot visually differentiate between titles and thumbnail descriptions. The list of topics or categories that the BVI users wish to browse is challenging to comprehend, as the thumbnail descriptions are addressed first without providing a clear notification when the title of the collection or topic is mentioned.

#### 4.2.5. Content: Difficulty Recognizing the Content of Images

**Figure 8:**
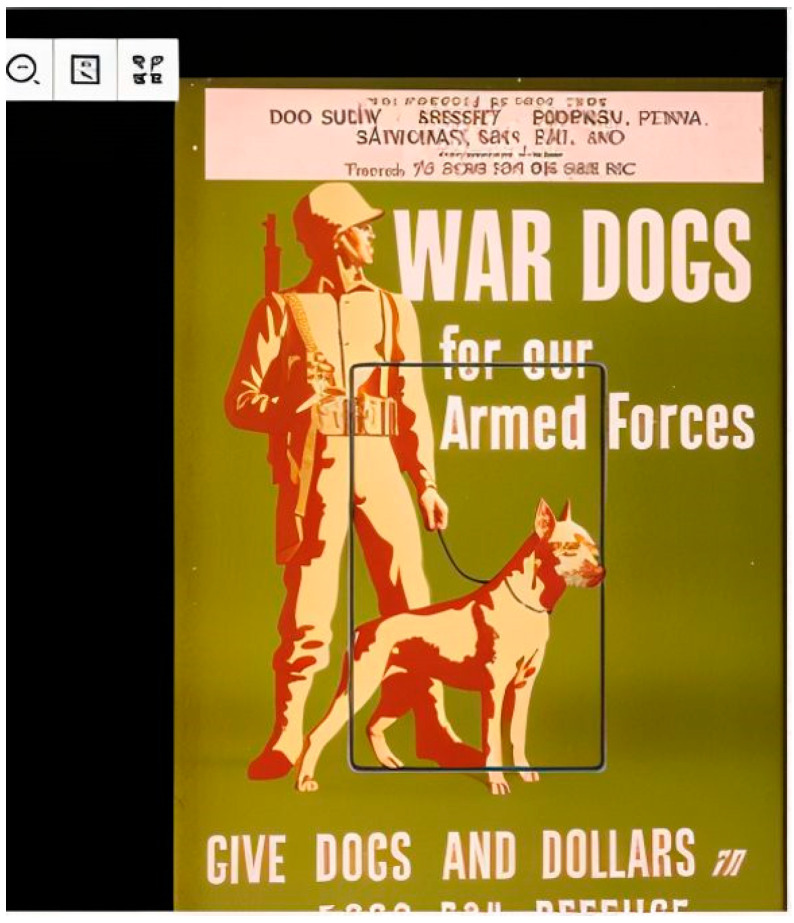
An example of difficulty recognizing the content of images.

This situation occurs when BVI users struggle to recognize the content of images (e.g., photographs, paintings, and illustrations). Images are mostly created using cameras, drawings, paintings, or relevant software. They can quickly convey visual information for illustrative or aesthetic purposes. Because of their visual nature, images are not friendly to BVI users, and thus, it is important for BVI users to rely on assistive technology (e.g., screen readers) to access such resources. However, BVI users were observed to have difficulty accessing the content of images when searching for information in DLs. For example, ID10-AL found the requested poster successfully when she was asked to find a World War II poster on war dogs in 1943 (Figure 8). However, she only heard the word “armed” from VoiceOver when trying to access the content of the found poster, making it challenging for her to obtain a comprehensive understanding of the content of the poster.

“Umm, oh, that’s the image there, but the only thing my VoiceOver said was the word armed. It just said like war dogs for armed forces image. And then it said armed. That’s all it says.”

(ID10-AL)

ID10-AL had difficulty recognizing the content of the found poster due to a lack of descriptions and ALT text for images and a lack of guidance for using assistive features (e.g., the image recognition feature of VoiceOver on iOS devices, the image description features of TalkBack on Android devices). It was found that descriptions as part of metadata did not provide detailed information on the visual characters (i.e., a soldier and a dog) presented in the poster. Meanwhile, the ALT text was inadequate to concisely convey information about important components of the poster. Another issue is a lack of instructions for BVI users about using relevant features of screen readers or other assistive technologies to access the content of images on their mobile devices.

#### 4.2.6. Content: Difficulty Recognizing the Content of Graphs

**Figure 9:**
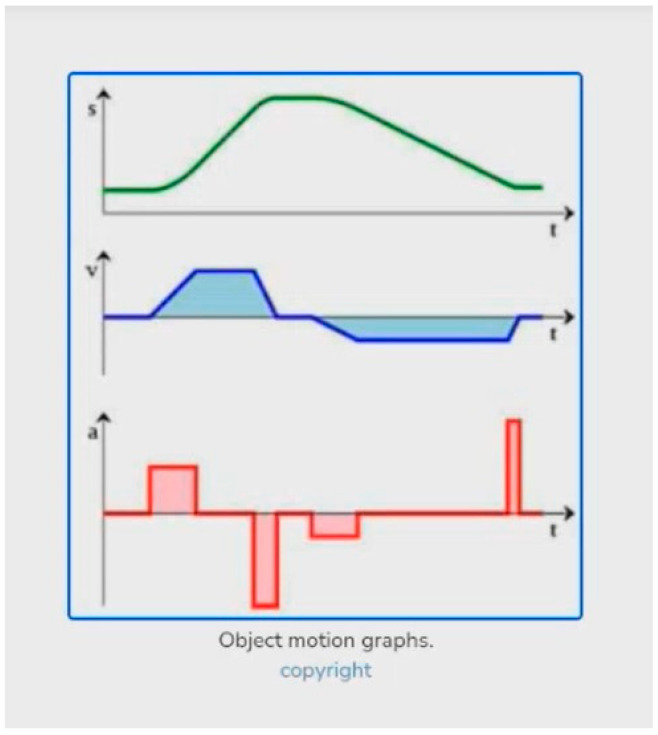
An example of difficulty recognizing the content of graphs.

This situation occurs when BVI users struggle to recognize the content of graphs. Visual documents such as graphs have a few issues with ALT texts and descriptions due to their unique characteristics. Graphs need more detailed descriptions than other types of visual items, such as images and videos, since they contain complex, multi-dimensional information such as points, trends, and axes. Moreover, graphs rely on various visual cues (e.g., color, shape, and position) to convey information, which makes it difficult for BVI users to understand their content with the screen reader. Specifically, one of the interviewees, AT18-OL, expressed difficulty obtaining details of the graphs due to a lack of descriptions. The participant was asked to find interactive items or graphics related to the relationships between position, velocity, and acceleration in the DL. While searching for the task-related items, he could not understand the graphs because they lacked descriptions (Figure 9).

“So, the first item that I think of will be a graph. However, this graph lacks a description. I can’t fully interpret because they’re just saying that these are graphs.”

(AT18-OL)

**Figure 10:**
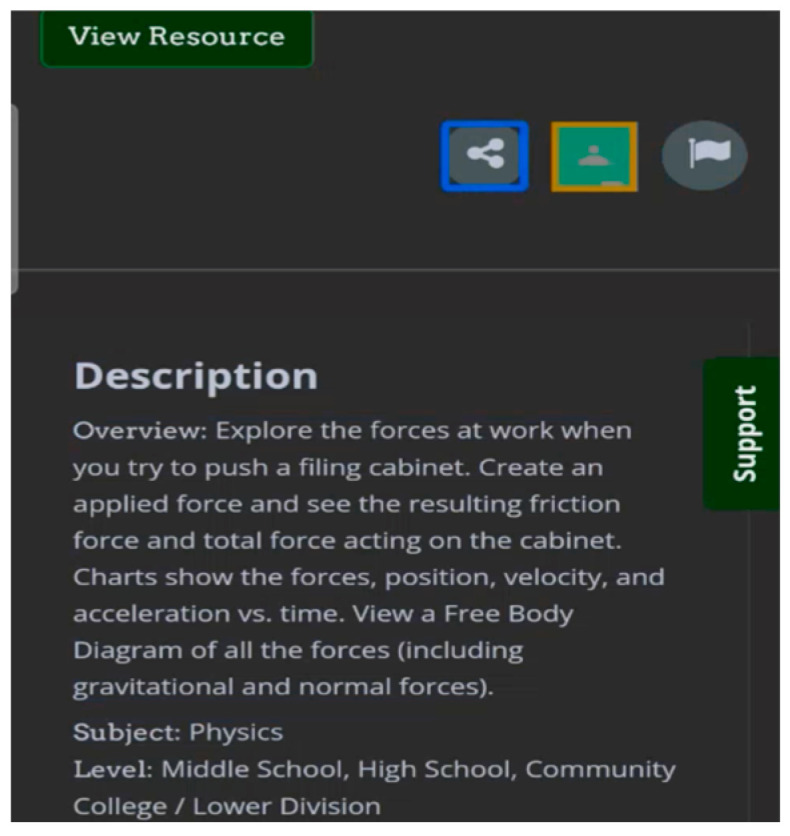
Another example of difficulty recognizing the content of graphs.

Even though another participant, AP15-LO, could access alternative descriptions (metadata) within the same task, it was still difficult to recognize the unique components of graphs, including legends and colors. AP15-LO was unable to grasp any specific information about the graph and could only access an overview of the graph (Figure 10).

“I can’t understand it. So, the description of it says that it’s a chart that shows forces, position, and velocity, but when I go into it, it’s not understandable.”

(AP15-LO)

AT18-OL added that Talkback did not give full descriptions, so he had to skip the graphs as he could not obtain more information. On the other hand, AP15-LO could obtain a description of the graph, but the explanation did not help her understand the graph. As shown in the examples, BVI users often encounter challenges when attempting to obtain details on graphs. One of the system factors causing these challenges is a lack of context-specific descriptions. Findings show that generalized descriptions without graphical components hampered users’ understanding. Another factor is the lack of guidance for image description options in mobile environments. Both iOS and Android offer image description options, such as VoiceOver recognition and automatic Talkback descriptions; nevertheless, some BVI users need assistance activating them in different applications and devices. These system factors lead BVI users to be confused or frustrated when they access specific types of visual documents, like graphs in DLs.

#### 4.2.7. Structure: Difficulty Interacting with Multilayered Windows

This situation occurs when a screen reader’s focus is not confined to a single window, allowing users to interact with multiple layers of windows that are grayed out. A pop-up window, which is visually evident for sighted users, may cause confusion for BVI users if not designed to support BVI users. If the pop-up window design allows screen readers to move outside of the window and interact with the content beneath it, BVI users may have difficulty recognizing that the content they are interacting with is not within the pop-up window.

**Figure 11:**
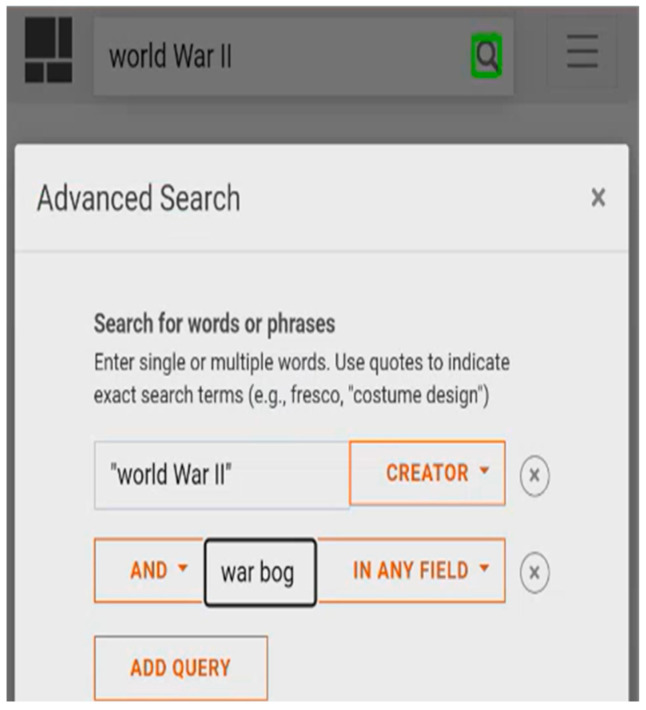
An example of difficulty interacting with multilayered windows.

For example, AP7-LA interacted with an advanced search feature designed as a pop-up window. For this task, AP7-LA was asked to find a poster on war dogs related to World War II. The initial search was conducted using the term “World War II”, yielding unsatisfactory results. To refine the search, she used the advanced search feature. After selecting all the refinements in the advanced search feature, she tried to move to the bottom of this feature window to execute the search. However, her screen reader focus navigated to the general search button without any feedback notifying her of her position. Although she believed she was interacting with the search button at the bottom of the pop-up window, she was interacting with the search button outside of the pop-up window. This led her to the results of a previous keyword search (Figure 11).

“I’m doing filtering…I wanted my filter…I wanna go to the other search…I went down to the bottom to touch on search. … I’m going back to my advance and I want to I wanna see. OK, I want to see. Umm. No, I took it out. I went back to my filters and it took my words.”

(AP7-LA)

The absence of constraints within pop-up windows caused the participant to have limited situational awareness. She mistakenly perceived that the advanced search feature had omitted her words from the search query because the search was, in fact, performed using the general search function instead of the intended advanced search. The lack of constraints designed for screen readers’ interaction with pop-up windows allowed this situation to occur, leading the participant to misunderstand her current situation. This could further complicate the overall search process as users are uncertain why their search did not work.

## 5. Discussion

This study reports partial results of a large-scale project regarding unique situations faced by BVI users when using DLs and corresponding design recommendations involving multiple types of mobile platforms and devices. The findings of the study have both theoretical and practical significance.

### 5.1. Theoretical Implications

Theoretically, this is one of the most comprehensive studies involving BVI users that help researchers better understand BVI users’ problems in their DL search processes. It investigated the help-seeking situations that 120 BVI users encountered in their interactions with six diverse DLs on four types of mobile devices. The findings of this study not only share some of the similar results from previous research but also yield some specific discoveries. This study has its contributions associated with features/elements, visual content, and structure. The seven help-seeking situations of DL visual interactions we reported in the study represent unique specific aspects of situations even though some of their broader issues might have been identified in previous research.

First, as to features/elements, while previous research has mainly focused on the challenges that BVI users encounter in visual content interactions [2,7,16,22,23,24,25,27], this study shows that features/elements posed the highest number of help-seeking situations (41.8%) for BVI users. Among the situations related to features/elements, this study echoes previous findings [2,22]. Icon-based features/elements, such as toggle-based search features, prevent BVI users from accessing and using these features/elements, thus disrupting their interaction process. Inadequate labels for the features/elements make finding them difficult for BVI users. Moreover, even when they find these features/elements, they have to take additional steps to use these features/elements.

Most importantly, this study identified several unique situations related to features/elements that prior research has not reported. For example, compared with other icon-based features/elements, video features are more complicated and dynamic. On the one hand, the lack of labels or inadequate labeling of video features makes it difficult for BVI users to make relevance judgments about the videos. On the other hand, video features present their progress using visual cues that BVI users cannot perceive and comprehend. Another unique finding is that BVI users cannot figure out paginated sections presented like a slide show and organized by unlabeled tabs. Paginated sections are unique features for DLs to promote specific collections or items. Active elements without specific labels cause confusion for BVI users. Difficulty distinguishing collection labels from thumbnail descriptions is a distinct situation in DLs. It reveals problems associated with two entities combined in a visual format without separation, perceivable by sighted users but not BVI users. This study raises the issue of how to consider users with different needs to understand this type of design.

One of the key reasons for these challenges is the small screen size. Mobile and tablet devices generally have smaller screens, which can lead to the limited input and interaction capabilities of mobile phones and tablets. Mobile devices with touch interfaces may be difficult for BVI users to interact with due to the reliance on gestures and small, hidden icons and the lack of physical buttons, which limits tactile feedback [16,19]. Also, some websites are not appropriately optimized to display properly on smaller screens, leading to poor user experience. In effect, information resources that are graphically oriented often result in restricted access for BVI users [47].

Second, help-seeking situations related to visual content are not unique in the mobile environment. Nevertheless, the uniqueness lies in the metadata of DLs and mobile screen readers’ image description features. Researchers have investigated the problems associated with images [7,16,22,23,24,25], graphs [16,27], and PDFs [26,28]. This study specifies the help-seeking situations related to images and graphs in the context of DLs. In addition to short ALT text, DLs typically also provide metadata for images and graphs, which can provide more detailed information than ALT text. However, some DLs have not presented adequate ALT text and descriptions, particularly for graphs. It is critical to describe a graph in a manner that BVI users can comprehend. The challenge is how to explain a graph effectively to BVI users with varying levels of domain knowledge who cannot see the graph. Moreover, mobile screen readers, such as VoiceOver and TalkBack, have image description features [6,19], but BVI users may not be aware of them.

Third, previous research has identified structure-related help-seeking situations related to overall structure in different systems or applications, such as health applications [18,27], camera applications [29], and DLs [2,30]. While prior research focused more on BVI users’ problems in understanding the overall structure, this study specifically highlights difficulty interacting with multilayered windows in DLs. This situation was also discovered in the context of online course platforms [31]. In both contexts, BVI users could not interact with pop-up windows. Instead, their foci were still on their original windows. This problem is devastating for BVI users because they need assistance before they can move forward.

Even though the situations were identified from the DL context, they could also occur in other systems or applications. The discovery of these situations is the first step for researchers to understand the ways in which designs hinder BVI users’ visual interactions with DLs and other systems. It sets up the foundations for researchers and practitioners to further enhance DL design and other systems to support BVI users.

### 5.2. Practical Implications

This section proposes design recommendations tailored to each situation. Additionally, AI implications for each of the situations are also introduced. The following paragraphs highlight design recommendations for the improvement of DL interfaces to deal with the identified help-seeking situations associated with features/elements, visual content, and structure.

Regarding design issues for features/elements, several help situations are caused by a lack of or inappropriate labels. Imaged-based icons represent the main problem for BVI users when accessing these features. For example, the toggle-based search feature is presented as an icon without an adequate label. A meaningful label to indicate the location of search boxes that can be captured by screen readers is critical for BVI users to find the search box. Of course, these features also need to be placed in easy-to-find locations. All captions, descriptions, and identifiers associated with search fields and interface elements should be clear, concise, and properly labeled and positioned in easily recognizable locations [7]. The same can also be applied to video features that are not clearly labeled. It is important to provide a predictable label for video-related features. Using the Accessible Rich Internet Applications (ARIA) attributes to provide clear and descriptive labels for all video controls is a good approach. These labels should describe the functionality of each control [48]. When a control is activated, feedback to confirm the action is needed. This can be achieved by changing the state of the button (e.g., from “Play” to “Pause”) and announcing the change via ARIA live regions. It is essential to use a consistent layout and design for video controls across a DL. This consistency helps BVI users predict where controls will be located and how they will function. AI can verify the correct application of ARIA to ensure that these elements function as intended [49,50]. Additionally, applications that allow alternative interaction methods, such as a motion gesture [51], can be introduced so BVI users can easily use video controls.

Even though labels are also associated with difficulty navigating items on paginated sections, the emphasis is on how to ensure labels for tabs are assigned to indicate their order. Feedback mechanisms could be implemented to announce the user’s current page and position within paginated sections (e.g., Page 2 of 5, item 3 of 10). Pagination controls (e.g., tabs and arrows) used to navigate users’ current position should be consistently placed and easy to access, with tactile feedback. AI could automatically generate and improve labels for various structural elements, such as buttons, tabs, and links, which pose challenges for BVI users if they are not properly labeled [34]. Labels and descriptions are closely related. To overcome the situation of difficulty distinguishing collection labels from thumbnail descriptions, we need to separate collection/item titles from thumbnail descriptions. When images are for decorative purposes, such as serving as a thumbnail, the image should have a null ALT text as recommended by the Web Content Accessibility Guidelines. Simultaneously, the image should be part of the text link to easily identify and increase the clickable area of an item [48]. However, if the thumbnails provide additional information that would be useful for BVI users, semantic HTML tags, such as <figure> for thumbnails and <figcaption> for descriptions, could be implemented to clearly define and separate these elements. At the same time, AI can also generate descriptive labels that clearly differentiate between collection titles and thumbnails [34].

In terms of design issues for visual content, two help-seeking situations are caused by a lack of descriptions, inadequate descriptions, or clear indications of how to access these descriptions. Difficulty recognizing the content of images is associated with BVI users being unable to find descriptions of images. Concise and informative descriptions need to be added as part of the metadata for all images in a DL, thereby conveying the essential information and context of the image. In addition, it is critical to provide ALT text that describes images [37,48]. Additionally, DLs can provide BVI users with instructions about using relevant features of screen readers or other assistive technologies to access the content of images on their mobile devices. Researchers have advised developers to implement automatic image captioning to help generate high-quality image descriptions using AI [49,52,53].

Compared with images, it is more difficult for BVI users to recognize the content of graphs because of their diverse formats and domains. Tools have been implemented to help BVI users understand the concept of charts [41] as well as create and access graphs [54]. For graphs that change dynamically, we can use ARIA attributes to update the ALT text dynamically as the image changes [48]. Integrating AI tools, like Envision AI, Lookout, or Seeing AI with DLs, helps users obtain auditory descriptions of graphical content. Envision AI excels in Optical Character Recognition, allowing it to read out text from images with high accuracy. Moreover, Envision AI can describe the contents of a scientific diagram or an architectural blueprint, providing BVI users with a deeper understanding of visual items [55].

In terms of structure, difficulty interacting with multilayered windows is associated with the existence of multiple windows that BVI users cannot detect and/or act upon. If possible, limiting the design of pop-up windows is the best solution to reduce the problem. If not possible, semantic HTML can be used to clearly convey the meaning and structure of web content for interpretation. Simultaneously, ARIA should be added to HTML elements to define regions of a webpage [48]. AI can also enhance the functionality of screen readers by providing context-specific information about a webpage’s structure. For example, “AccessiBe”, powered by machine learning, automatically identifies and informs BVI users of structural challenges within web pages, providing real-time feedback and adjustments to enhance accessibility [56].

## 6. Conclusions

We conducted a comprehensive user study with 120 BVI users to investigate the help-seeking situations they face when interacting with DLs on various types of mobile devices, including iPhones, iPads, Android Phones, and Android Tablets. Using multiple data collection methods, including questionnaires, think-aloud protocols, transaction logs, and interviews, we identified various help-seeking situations encountered by BVI users and associated factors causing these situations.

This paper reports seven typical help-seeking situations associated with visual interactions categorized into three groups: features/elements (e.g., toggle-based search features), content (e.g., graphs), and structure (e.g., multilayered windows). Based on our findings, we provided design recommendations to enhance the accessibility and usability of DLs for BVI users. These include the use of clear and consistent labeling for visual elements and features, providing helpful information for interacting with visual content, and the use of simple page layout and site structure. Also, the use of emerging AI technologies can help verify and improve the design of DLs for BVI users. As part of an ongoing project, this paper focuses on identifying specific help-seeking situations related to visual interactions. Our immediate next steps will involve a quantitative analysis of the relationships between the participants’ demographic characteristics, types of mobile platforms and devices, and types of help-seeking situations encountered.

While our study offers valuable insights, it is important to acknowledge certain limitations. First, although the six DLs we selected represent diverse types of DLs in terms of their content domains (e.g., history, art, and science), formats (e.g., image, video, and mathematical formulas), and nature of hosting organizations (e.g., the federal government and non-profit organization), our findings may not be fully generalizable. Second, the online data collection method using virtual conferencing tools, which allowed us to reach a wide range of BVI users across the nation, might have hindered the natural interactions of BVI users with DLs. Our future research will involve comparing the similarities and differences of help-seeking situations across more diverse DLs. Also, study designs using face-to-face interactions can be considered to identify more detailed issues with the accessibility and usability of DLs. These findings will inform the refinement of the design for DLs and other types of IR systems on mobile platforms.

## Figures and Tables

**Table 1:** Demographic information of participants (N = 120).

	Category	Number of Participants
Age	18–29	29
	30–39	41
	40–49	27
	50–59	13
	>59	10
Gender	Female	60
	Male	59
	Non-binary	1
Race	White	54
	Black	24
	Asian or Pacific Islander	19
	Hispanic or Latino	12
	Other	11
Education	High school and Associate	30
	Bachelor	46
	Master	33
	Doctoral or professional degree	8
	Prefer not to answer	3
Vision condition	Blind	80
	Visually impaired	40

**Table 2:** Task types and examples.

Task Type	Task Duration	Example (DL: Task Information)
Orientation task	10 min	LoC, DPLA, HathiTrust, ArtStor, OER Commons, and the National Museum of African American History and Culture: You will have 10 min to explore the features and functions of this digital library. Please talk continuously about your thoughts and actions in relation to your interactions with the digital library during this task including its structure, features, content, format, search results, etc. Please specify your intentions for each action, the problems you encountered, and your solutions.
Specific search	15 min	ArtStor: Find a World War II poster on war dogs in 1943. Find out the name of the repository that houses it. What were the two figures portrayed in the poster?
Exploratory search	15 min	OER Commons: Find interactive items or graphics related to relationships between position, velocity, and acceleration. Each should represent different formats (e.g., interactive item, image, and text) or content (e.g., the relationship between velocity and acceleration, the relationship between position and velocity) of this search topic.

**Table 3:** Coding scheme of seven help-seeking situations.

Category	Type of Situations	Definition
Features/elements	Difficulty finding a toggle-based search feature	A situation that arises when BVI users have difficulty finding a search box due to the unrecognizable toggle-based search feature.
	Difficulty understanding a video feature	A situation that arises from difficulty interpreting the functionality of or executing a video-related feature due to inappropriate labeling.
	Difficulty navigating items on paginated sections	A situation that arises from difficulty selecting an item on paginated sections due to inappropriate labels of active elements.
	Difficulty distinguishing collection labels from thumbnail descriptions	A situation that arises from difficulty recognizing and comprehending collection titles due to the unseparated collection titles and ALT text for thumbnails.
Content	Difficulty recognizing the content of images	A situation arises from difficulty obtaining details of images due to a lack of support for providing specifics of images.
	Difficulty recognizing the content of graphs	A situation that arises from difficulty obtaining details of graphs due to a lack of support for providing specifics of graphs.
Structure	Difficulty interacting with multilayered windows	A situation that arises from difficulty focusing on an active window due to the poor design of pop-up windows.

## Data Availability

The data presented in this study are available on request from the corresponding author due to the project’s ongoing status.
